# Comparison of H7N9 and H9N2 influenza infections in mouse model unravels the importance of early innate immune response in host protection

**DOI:** 10.3389/fcimb.2022.941078

**Published:** 2022-08-12

**Authors:** Cuisong Zhu, Miaomiao Zhang, Weihui Fu, Yongquan He, Yu Yang, Linxia Zhang, Songhua Yuan, Lang Jiang, Jianqing Xu, Xiaoyan Zhang

**Affiliations:** ^1^ Department of Scientific Research, Shanghai Public Health Clinical Center, Shanghai, China; ^2^ Department of Pathology, Institute of Clinical Science and Shanghai Key Laboratory of Organ Transplantation, Shanghai, China; ^3^ Human Disease Genes Key Laboratory of Sichuan Province and Institute of Laboratory Medicine,Sichuan Academy of Medical Sciences and Sichuan Provincial People's Hospital, University of Electronic Science and Technology of China, Chengdu, China; ^4^ Department of Pathology, Fudan University Shanghai Cancer Center, Shanghai, China

**Keywords:** H7N9, H9N2, pathological characteristics, C57BL/6 mice, lung

## Abstract

The outcome of infection with influenza A virus is determined by a complex virus-host interaction. A new H7N9 virus of avian origin crossed the species barrier to infect humans, causing high mortality and emerged as a potential pandemic threat. The mechanisms underlying the virulence and pathogenicity of H7N9 virus remains elusive. H7N9 virus originated from a genetic assortment that involved the avian H9N2 virus, which was the donor of the six internal genes. Unlike the H7N9 virus, the H9N2 virus caused only mild phenotype in infected mice. In this study, we used the mouse infection model to dissect the difference in the host response between the H7N9 and H9N2 viruses. Through analyzing transcriptomics of infected lungs, we surprisingly found that the H9N2 infection elicited an earlier induction of innate immunity than H7N9 infection. This finding was further corroborated by an immunohistochemical study demonstrating earlier recruitment of macrophage to the H9N2-infected lung than the H7N9-infected lung, which could occur as early as 6 hours post infection. In contrast, H7N9 infection was characterized by a late, strong lung CD8+ T cell response that is more robust than H9N2 infection. The different pattern of immune response may underlie more severe lung pathology caused by H7N9 infection compared to H9N2 infection. Finally, we could show that co-infection of the H9N2 virus protected mice from the challenge of both H7N9 and PR8 viruses, thereby strengthening the importance of the induction of an early innate immunity in the host’s defense against influenza infection. Collectively, our study unraveled a previously unidentified difference in host response between H7N9 and H9N2 infection and shed new insight on how virus-host interaction shapes the *in vivo* outcome of influenza infection.

## Introduction

Since the first virus strain was identified in 1930s, influenza A viruses (IAVs) continue to pose a threat to humans. The IAV challenge, at least in part, stems from the fact that multiple hosts exist, including a variety of avian species, pigs, and humans, and new viruses can emerge from reassortment when two or more viruses infect the same cell. Normally the species barriers would prevent IAV’s spillover from animal to human. However, such a barrier could be broken, resulting in human exposure to new viruses to which they do not have immunity, and consequently can lead to high mortality and even a pandemic if the virus evolves into acquiring human-to-human transmission ability. Most recently, a case was found in a novel avian IAV of H7N9 subtype (abbreviated as H7N9 throughout this paper), which was first reported to cause severe human respiratory infection with high fatality rate in Shanghai and Anhui Province, China, in March 2013 (Gao et al., 2013; Wang et al., 2017). Phylogenetic analysis revealed that H7N9 is a reassortment virus having hemagglutinin (HA) and neuraminidase (NA) genes derived from Eurasian avian influenza viruses (AIVs) and six internal genes from avian H9N2 genotypes (Ku et al., 2014). The death rate of H7N9-infected patients is as high as 40% (Wang et al., 2017; Wu et al., 2020), whereas H9N2 infections only cause mild symptoms in humans (Song and Qin, 2020). Recent research has also indicated a high level of genetic compatibility between H9N2 and H7N9 viruses, raising the likelihood that there could be continuous reassortment between H7N9 and H9N2 viruses co-circulated in poultry and that internal gene replacement could convert the H9N2 virus into a new threat to public health (Pu et al., 2015; Zhang et al., 2020).

The immune response of the host, comprised of two arms, innate immunity and adaptive immunity, is crucial in determining disease pathogenesis caused by viral infection and is the basis for the development of control strategies (Koutsakos et al., 2019). While a potent immune response is essential for effective viral inhibition, it must be fine-tuned, as an excessive and/or prolonged inflammatory response has been correlated to exacerbation of tissue damage both in human and animal models. This double-edged effect was clearly demonstrated in the case of H5N1 virus, which like H7N9, is of avian origin and causes severe clinical manifestation with a high mortality rate. H5N1 infections were associated with dysregulated elevation of proinflammatory cytokines, referred to as a cytokine storm, which is widely regarded as the major cause of widespread pulmonary tissue damage associated with H5N1 infection (Kash et al., 2006; Wu et al., 2020).

There have been several published studies on the characterization of H7N9 virus in mice. These studies revealed that H7N9 virus caused more severe phenotype than H9N2 virus and seasonal H3N2 influenza virus, which correlates to increased infectivity and elevated induction of proinflammatory cytokines (Belser et al., 2013; Mok et al., 2013; Meliopoulos et al., 2014; Bi et al., 2015). Of interest, the host response and the outcome of infection also varied with the mouse strain used, as C57BL/6 mice exhibited more severe phenotype than BALB/c mice, which was accompanied by a different profile of proinflammatory cytokines (Zhao et al., 2014). It was found that H7N9 infection induced significant elevation of proinflammatory cytokines at a level higher than the duck-derived H9N2 virus and the H9N2 virus of avian origin, but lower compared to that induced by the H5N1 infection (Mok et al., 2013). Thus, despite causing similar morbidity and mortality in humans, H7N9 and H5N1 may differ in the disease-causing mechanisms.

Our group has adopted a comparative approach to understanding the mechanisms underlying mammalian adaptation and pathogenesis of the H7N9 virus using H9N2 as a counterpart. Through performing bulk transcriptomic profiling of lung tissues from virus-infected mice, we recently identified IFN-κ as one of the earliest Type I IFNs induced by the H9N2 infection whereas this early induction was not featured in response to H7N9 infection (He et al., 2020). This finding prompted us to further dissect the difference between the host immune response in the H7N9 infection and the H9N2 infection using the mouse model; the results of such exploration are presented here.

## Materials and methods

### Viruses

A/Shanghai/4664T/2013(SH/4664) H7N9 virus (Genbank accession number: KC853225.1-KC853232.1) was conserved at a Biosafety Level 3 (BSL3) lab at Shanghai Public Clinical Center. A/Chicken/Shanghai/F/98 (CK/F98) H9N2 virus (Genbank accession number: AY253750.1-AY253756.1, AY743216.1) was conserved at Shanghai Veterinary Research Institute, Chinese Academic of Agricultural Sciences.

### Animal studies

Eight- to ten-week-old female C57BL/6 mice (B&K Universal Group Limited, Shanghai, China) were maintained under specific pathogen-free conditions at the animal facilities of Shanghai Public Health Clinical Center. For the animal study comparing virulence of H7N9 versus H9N2, mice were randomly grouped, anesthetized *via* intraperitoneal injection of ketamine (100mg/kg), and then intranasally inoculated with a dose of 1× 10^6^ EID50 (50% egg infectious dose) virus, or PBS in a volume of 50μl. The infected mice, where indicated, were divided into subgroups for different analyses; for the assessment of morbidity and mortality, the mice were monitored for survival and body weight changes over a 14-day observation period. Mice with 30% or more body weight loss were recorded dead and humanely euthanized. For the co-infection experiment, mice were intranasally inoculated with 1x10^4^ TCID50 of H7N9/PR8, or 1.7x10^7^ EID50 of H9N2, or both viruses in a 50µl volume. All the animal-related experiments were carried out in a BSL3 facility at Shanghai Public Clinical Center. This study was performed in accordance with Home Office guidelines and were approved by the Shanghai Public Health Center Local Ethical Committee.

### Histopathological and immunohistochemical staining

Mice were sacrificed at 6 hours, day 1, day 2, day 3, and day 7 after the virus challenge for collection of lung tissues. Lung tissues were fixed with 4% paraformaldehyde and then embedded in paraffin. Five-micrometer sections were cut and stained with H&E. The pathology scores were calculated following a methodology we previously published (He et al., 2020). For immunohistochemical (IHC) assays, paraffin sections of the lung were de-waxed and then subjected to heat treatment in citrate buffer, followed by quenching of endogenous peroxidase activity using 0.3% H2O2 in methanol. Sections were blocked for 1 hour (hr) with Fc Receptor Blocker and then incubated overnight at 4°C with F4/80 antibody (CST No.70076, 1:500). Antibody binding was detected using ZSGB System reagents (ZSGB, Beijing). After counterstaining with haematoxylin, the slides were incubated with CD4-specific antibody (Abcam No. ab183685, 1:1000) and CD8-specific antibody (LSBio No.LS-C43572, 1:200), and multiplexed immunofluorescence staining was then performed using Opal 7-color Manual IHC Kit (Akoya,USA). The stained slides were scanned by TissueFAXS 200 (TissueGnostics). The acquired images were analyzed by Strata Quest software to assess the number of F4/80+, CD4+, CD8+ cell and the inflammatory infiltration areas.

### Extraction of total RNA and microarray analysis

Each lung from the H7N9/H9N2 infected mice was mechanically and ultrasonically homogenized at 4°C in 1.2ml RNAzol (MRC, OH, USA). Total RNA was extracted from homogenized tissue according to the protocol supplied by the manufacturer. The resulting RNA preparations were analyzed on a 2100 Bioanalyzer (Agilent Technologies, Waldbronn, Germany) to ensure quality and integrity. For microarray hybridization, 500ng of RNA were applied to Cy3-labelling reaction using the one-color Quick Amp Labelling protocol and the labeled cDNAs were hybridized to Agilent’s mouse 8 x 60 k microarrays followed by detection using the Agilent Scanner G2505C (Agilent Technologies). The microarray data were analyzed by RBR embedded in Excel and cluster analyses were performed using MEGA5. The microarray data were deposited in Gene Expression Omnibus (GEO) under accession number GSE142709.

### Quantitative RT-PCR

Total RNA was isolated from homogenized lung tissue using an RNA isolation kit (Qiagen, Valencia, CA). Reverse transcription was performed using oligo (dT) primer and the Reverse Transcription System (Promega, Madison, WI), and SYBR green-based real-time PCR was performed on the resulting cDNAs using Mastercycler reprealplex real-time PCR system (Eppendorf, Germany). The data were analyzed by the REALPLEX2.2 software. The gene-specific primers used in this study were: mCXCL10: F-5’-GGTCTGAGTCCTCGCTCAAG-3’, R-5’-GTCGCACCTCCACATAGCTT-3’; mIL1β: F-5’-GAAATGCCACCTTTTGACAGTG-3’, R-5’-CTGGATGCTCTCATCAGGACA-3’; mIL6: F-5’-CTGCAAGAGACTTCCATCCAG-3’, R-5’-AGTGGTATAGACAGGTCTGTTGG-3’; mTNFα: F-5’-CATCTTCTCAAAATTCGAGTGACAA-3’, R-5’-TGGGAGTAGACAAGGTACAACCC-3’; mGAPDH: F-5’-TGGCCTTCCGTGTTCC TAC-3’, R-5’-GAGTTGCTGTTGAAGTCGCA-3’.

### Lung viral titer determination and RNA *in situ* hybridization

Lung viral titer determinations, using 9-day-old embryonated eggs, were performed following our published procedure (Zhang et al., 2020). For analyzing viral RNA expression in the lung section by RNA *in situ* hybridization, we chose viral nucleoprotein (NP) RNA as target RNA for detection because of its high expression in infected cells and high conservation among different virus strains. An NP-specific probe, namely Probe-NP, is customized to target 236-1367 region of the NP gene of the influenza virus A/Shanghai/1/2013 (H7N9) (GenBank: KF609528.1). Sequence comparison confirmed that the targeted NP gene sequence is highly conserved in the H9N2 virus. The preparation of slides and RNA *in situ* hybridization using RNAscope reagents (Advanced Cell Diagnostics) were performed as described previously (He et al., 2020).

### Statistical analysis

All the statistical analyses were performed using the GraphPad Prism 5 software (GraphPad Software, Inc. La Jolla, CA, USA). Data were expressed as mean ± SEM. One-way analysis of variance (ANOVA) test was applied when comparing more than two groups. A *P* value of ≤0.05 was considered statistically significant

## Results

### Comparison of the virulence of the H7N9 and H9N2 viruses in C57BL/6 mice

**Figure 1:**
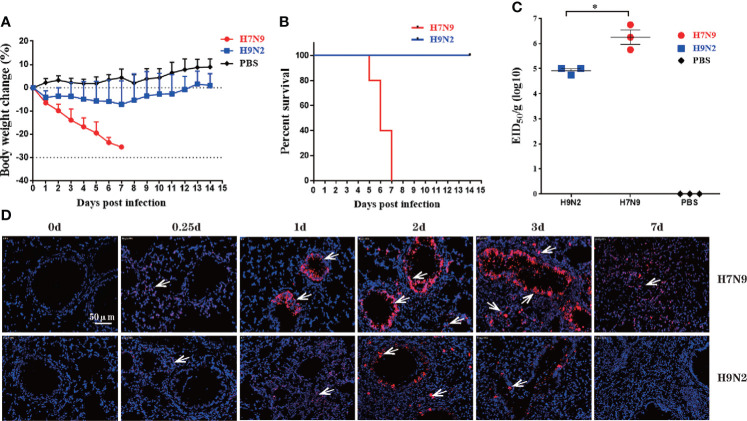
H7N9 virus is more virulent than H9N2 virus in C57BL/6 mice. Mice were intranasally inoculated with a single dose of 10^6^ EID_50_ of the H7N9 virus or the H9N2 virus. **(A)** Weight loss during a 14-day observation period. **(B)** Survival curve during a 14-day-observation period. Mice that lost >30% of their pre-inoculated weight were regarded as dead and euthanized. **(C)** Virus titers in mice lungs, as determined by EID_50_ using MDCK cells. **(D)** Representative images of RNAscope *in situ* hybridization (ISH) showing virus NP expression in lung. Each red dot represents a single NP RNA molecule, further indicated by white arrow, with nuclei counterstained by DAPI. Data are expressed as mean ± SEM.*P < 0.05 by one-way ANOVA.

We used a mouse infection model to compare the virulence of the H7N9 virus versus the H9N2 virus. To this end, C57BL/6 mice were intranasally inoculated with PBS (mock) or a dose of 1 × 10^6^ EID50 of either the H7N9 or H9N2 virus, and monitored over a period of 14 days for survival and weight loss. Part of the group was subjected to lung viral load determination at 4-days post infection (dpi) by titration in embryonated eggs. The H7N9 challenged group showed a sustained weight loss starting from 1 dpi, whereas the H9N2 challenged group experienced a moderate weight decrease during the first 7 days and regained weight afterwards (**Figure 1A**). Accordingly, all the H7N9 infected mice died before 7 dpi whereas the H9N2 group all survived to the end of the observation period (**Figure 1B**). The average lung viral titer measured at 4 dpi for H9N2 group was approximately 20-fold lower than that of the H7N9 group (8.32 × 10^4^ EID50/g versus 1.78 × 10^6^ EID50/g) (**Figure 1C**).

To further observe the progression of virus infection, we employed RNA *in situ* hybridization technology (RNAscope) to assess the dynamic distribution of virus-infected cells, indicated by the presence of viral NP RNA, in the lung. Cells positive for NP RNA (NP-positive cells) could be detected in both bronchi and alveoli of the lung section from H7N9 infected mice 6 hours post infection (0.25 dpi) and saw a marked increase in the number over 1-3 dpi, followed by a sharp decline observed at 7 dpi. The number of NP-positive lung cells were significantly lower in samples from the H9N2 group, compared to those of H7N9 group at all time points until becoming undetectable at 7dpi (**Figure 1D**). Thus, the RANscope data were consistent overall with lung viral titer measurements, confirming higher replication capacity of the H7N9 virus than the H9N2 virus. Collectively, these results demonstrated that the H7N9 virus was more virulent than the H9N2 virus in C57BL/6 mice.

### Comparative analysis of gene expression dynamics in the lungs of infected mice

**Figure 2:**
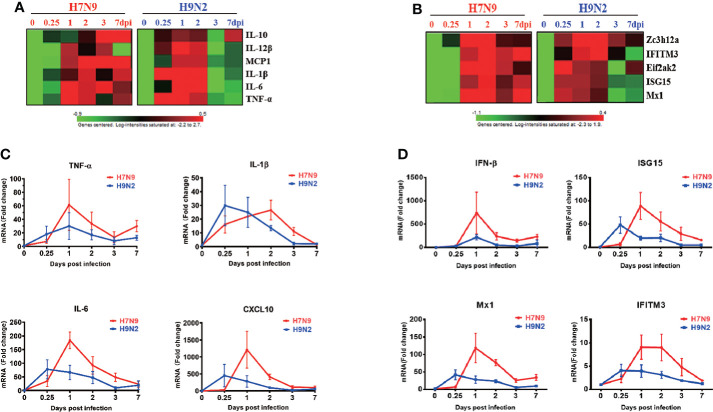
Comparative analysis of gene expression dynamics in the lungs of infected mice. Total RNA of lung tissues harvested at 0,0.25,1,2,3,7 dpi from H7N9- or H9N2-infected mice were subjected to microarray-based transcriptome analysis. The microarray data were used for analyzing the expression of proinflammatory cytokines and anti-viral ISGs, expressed as heatmaps of relative gene expression after normalization to the value of mock-infected mice **(A, B)**. Real-time RT-PCR was performed to validate the microarray analysis (n=3 per time point), with the results shown respectively for cytokines **(C)** and anti-viral ISGs **(D)**.

To gain insight into host responses against the H7N9 and H9N2 viruses, we performed a microarray-based profiling of RNA extracted from lung tissues collected at a series of time points post virus challenge from H7N9- and H9N2-infected mice. Our initial analysis of this data revealed a significant difference between H9N2 and H7N9 infection in the induction of one type I interferon, namely IFN-κ, which was then demonstrated as a broad inhibitor of influenza replication *via* the IFNAR-MAPK-Fos-CHD6 axis (He et al., 2020). We further utilized this data to interrogate other aspects of immune response in the lung by focusing on genes indicative of immune status. The analyses revealed that relative to H7N9 infection, H9N2 infection was characterized by an earlier induction of immune response in the lung, as evidenced by prompt induction of interferon-induced genes and proinflammatory cytokines with key roles in the host’s response to influenza infection, including IL-10, IL-12β, MCP1, IL-1β, IL-6, and TNF-α, which occurred as early as 0.25 dpi (**Figures 2A, B**). Conversely, despite later initiation, the H7N9 infection-induced upregulation of proinflammatory cytokines appeared to be generally more sustainable with higher peak value as compared to that associated with H9N2 infection. These findings were further verified by measurements of representative cytokines/chemokines and ISGs using quantitative RT-PCR (**Figures 2C, D**). Interestingly, the earlier induction by H9N2 infection did not occur to IFN-β, which rather showed more upregulation in response to H7N9 infection, sharply rising to a peak at 1 dpi from a base level of 0.25 dpi and then dropping precipitously. The uncoupling between IFN-β and ISGs is consistent with the early ISG response to H9N2 infection and may be mediated by IFN-κ (**Figure 2B**). Thus, even with a lower viral replication, the H9N2 virus was able to induce an antiviral/inflammatory state in the infected lung faster than H7N9 virus, which might contribute to a better host protection.

### Comparative analysis of lung pathology and immune cell infiltration

**Figure 3:**
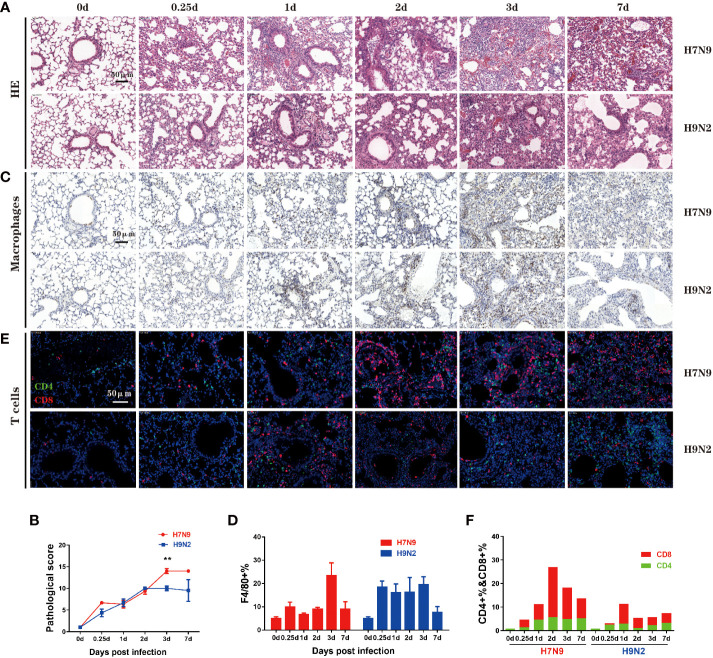
Comparative analysis of lung pathology and immune cell infiltration. **(A)** Lung tissue harvested at 0,0.25,1,2,3, and 7 dpi. from mice that had been infected H7N9 or H9N2 avian influenza virus was stained with H&E. **(B)** Quantified pathological scores derived from the calculation of lung histology data. **(C)** Immuno-histochemical detection of total macrophages in the lung sections by staining with anti-F4/80 antibody, with nuclei counterstained by hematoxylin. Brown signals indicate detected macrophages. **(D)** Summation data of the measurements of lung macrophages. **(E)** Immunofluorescence detection of CD4+ plus CD8+ T cells in the lung sections by sequential staining with anti-CD4 and anti-CD8 antibodies, with nuclei counterstained by DAPI. Red and green signals indicate detected CD8+ and CD4+ T cells, respectively. **(F)** Summation data of the measurements of CD4+ and CD8+ T cells in the lung. n=3 mice for each group per time point. **P < 0.01 by unpaired t test.

Next, we evaluated the lung pathology and immune cell infiltration during the course of infection. The degree of lung tissue injury was assessed using HE staining. The pulmonary pathological changes of mice infected with H7N9 were severe at 3 dpi and continued to worsen until death; the main injuries were characterized by massive red blood cell exudation and serious bronchial and bronchiolar damage (**Figure 3A**, upper panel). In contrast, the histopathology changes in the lung of H9N2-infected mice included alveolar wall thickening and inflammatory cell infiltration, which were most evident at 2 dpi. During subsequent days, although the injury of the lung tissue persisted, the inflammatory infiltration was reduced and consequently, the red blood cell exudation was significantly reduced at 7 dpi (**Figure 3A**). The progression of lung damage was further appraised by a pathological score curve, verifying that additional severe lung pathology was induced by the H7N9 infection compared to H9N2 infection at both 3 dpi and 7 dpi (**Figure 3B**).

The immune cell infiltration was assessed by immunohistochemical staining, focusing on two important immune cell types, namely macrophage and T cell. Macrophages play multiple functions in antiviral immune responses, including antigen recognition and secretion of interferons. After H7N9 infection, the frequency of lung macrophages moderately increased within 2 dpi before climbing to a peak level at 3 dpi which was significantly higher than the pre-infection (normal) level, and then underwent a sharp decline by 7 dpi (**Figures 3C, D**). In contrast, a substantial increase in the frequency of lung macrophages was observed after H9N2 infection by 0.25 dpi, which was maintained through 3 dpi before rapidly disappearing by 7 dpi (**Figures 3C, D**). Thus, H9N2 infection triggers a lung macrophage response more promptly than H7N9 infection, which is in accordance with the above bulky lung transcriptomics analysis revealing very early induction of interferon and proinflammatory cytokines by the H9N2 infection that did not occur with the H7N9 infection.

As one of the two arms of the adaptive immunity, T cells are critical for virus clearance. Previous analyses of human H7N9 patients indicated an early robust CD8+ T cell response is closely correlated with rapid recovery (Wang et al., 2015), whereas CD4+ T cells can directly contribute to viral clearance by cytokine secretion and provide help for B cells and CD8+ T cells (Kervevan and Chakrabarti, 2021). Our measurement identified 1dpi as a demarcation point of the two phases according to the difference between H7N9 and H9N2 infections in terms of lung CD8+ T cell response. Before 1 dpi, no significant difference appeared between the H7N9 and H9N2 infection. In fact, H9N2 induced the highest frequency of lung CD8+ T cells at 1 dpi over the course of infection, which was comparable to or even higher than that shown by H7N9 infection at the same time point. After 1 dpi, H7N9 infection was characterized by an elevation of induced CD8+T cell response, which was not observed with H9N2 infection. The CD8+ T cell response induced by H7N9 infection reached a peak level at 2 dpi and then gradually decreased afterwards, with the level at 7 dpi being still higher than the peak level observed with the H9N2 infection. The H7N9 infection was also associated with a generally stronger CD4+ T cell response than the H9N2 infection. Interestingly, this response did not have the dynamic feature of the lung CD8+ T cell response, showing a relatively stable level up to 7 dpi since showing a clear upregulation between 0.25-1 dpi (**Figures 3E, F**). Collectively, these immuno-histochemical studies highlighted a marked difference between H7N9 and H9N2 infections in eliciting lung macrophage and CD8+ T cell response in terms of both the timing and magnitude.

### An experimental utilization of H9N2 raised early innate immunity to protect against heterologous influenza challenge

**Figure 4:**
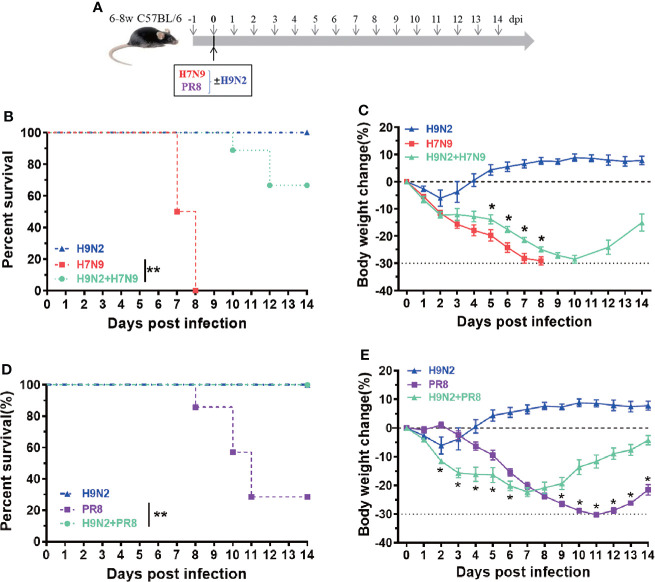
An experimental utilization of H9N2-raised early innate immunity to protect against heterologous influenza challenge. **(A)** The schematic diagram of a co-infection experiment. **(B, C)** Comparison of survival **(B)** and body weigh change **(C)** among mice infected with H7N9 alone, H9N2 alone, or a combination of H7N9 and H9N2. **(D, E)** Comparison of survival **(D)** and body weigh change **(E)** among mice infected with PR8 alone, H9N2 alone, or a combination of PR8 and H9N2. n=7-10 for each group, Data are expressed as mean ± SEM. *P < 0.05 by t test for body weight change at indicated time point between H7N9/PR8 infected group and H7N9/PR8+H9N2 co-infected groups. **P < 0.01 by Log-rank (Mantel-Cox) test for survival analysis.

To evaluate the importance of induction of early immune response in host protection, we designed a mouse co-infection study, in which the H9N2-induced early immunity was imposed against lethal H7N9 infection or PR8 infection. To this end, the mice were intranasally challenged with 1 x 10^4^ TCID50 of either H7N9 or PR8 virus, alone or in mixture with 1.7 x 10^7^ EID50 of H9N2 virus, and subsequently monitored for survival and weight loss (**Figure 4A**). Compared to the animal group with a single H7N9 infection, that all died within 9 days post challenge, a majority of the experimental group receiving the mixed infection survived at the end of the 14-day observation period (**Figure 4B**). This H9N2-mediated protective effect was also reflected in weight loss curves (**Figure 4C**). A protection was also observed in the co-administration of PR8 virus with the H9N2 virus for challenging mice; the infected animals exhibited better survival than those infected with PR8 and they also showed significantly better weight gain from 9 dpi, despite displaying more weight loss in the earlier period (**Figures 4D, E**). Collectively, these results further support our view that early innate immunity, once activated/primed, could afford the host a more effective antiviral defense against invading influenza viruses.

## Discussion

In this study, we compared the H7N9 and H9N2 influenza infections in the mouse model. We observed that, with the same challenging dose, H7N9 infection caused complete lethality before day 8 while the H9N2-infected group only initially exhibited mild weight loss before regaining normal weights and attaining full recovery by day 7. Such a difference in the outcome of infection was correlated to higher lung viral titers associated with the H7N9 infection compared to the H9N2 infection. Microarray-based analyses of gene expression in the lung surprisingly revealed an early induction of innate immunity by the H9N2 infection relative to the H7N9 infection, evidenced by the upregulation of ISGs, immune cell markers and proinflammatory genes. In accordance with this finding, we further showed that macrophages were recruited to the lung of H9N2-infected mice as early as 6 hrs post infection while the clear enrichment of macrophage in the lung was only observed for H7N9 infection on day 3. The lung macrophage response for both infections were subdued by day 7. In contrast, the H7N9-infected lung exhibited a more robust CD8+ T cell response than the H9N2-infected lung, peaking at day 2 and declining afterwards. The different pattern of immune response may correspond with the H7N9 infection which produced more severe lung pathology than the H9N2 infection. Finally, we could show that co-infection of the H9N2 virus led to an effective protection against both H7N9 and PR8 viruses, thereby strengthening the importance of an early innate immunity in the host’s defense against influenza infection. Collectively, our study unraveled a previously unidentified difference in the host’s response between the H7N9 and H9N2 infections: the H9N2 infection triggered an earlier innate immunity in the lung than the H7N9 infection. We proposed that such early engagement of host innate immunity, along with intrinsic weak infectivity in mammalian cells, permits an effective control of H9N2 in infected mice. It is tempting to speculate that the H7N9 virus evolves a strategy to avoid triggering early immune response and thus allows more effective virus propagation in the lung until the later arrival of adaptive immunity, which consequently contributes to lung pathogenicity as a collateral effect of viral clearance.

The study presented here is distinguished from previous mouse studies on H7N9 infection in that our analyses covered very early time points post infection starting from 6 dpi., which were previously neglected. Such an expanded investigation led us to a surprising finding that, relative to the H7N9 infection, the H9N2 infection triggered an early, while transient, innate immune response in infected lung in terms of both interferon-mediated antiviral signaling and the production of proinflammatory cytokines. We further corroborated this finding by demonstrating earlier recruitment of macrophages in the H9N2-infected lung compared to the H7N9-infected lung. In fact, we have used our microarray data to assess the expression dynamics of two monocyte-expressing chemokine receptors, namely CCR2 and CXCR4, which play key roles in mobilizing monocytes into lung in response to their respective ligand, CCL2 (MCP-1) and CXCL12. The analyses revealed that, compared to the H7N9 infection, the H9N2 infection induced an earlier induction of both CCR2 and CXCR4 transcripts, similar to that of CCL2 (**Figure 2A**) in the lung (data not shown). Thus, these findings, in line with the notion that the CCL2-CCR2 axis drives the lung migration of monocyte, supported the infiltrated monocytes as one if not the only source of macrophage accumulation in the lung responding to H9N2 infection. A major limitation of this study is that we had not measured the abundance of neutrophiles, which have well documented roles in influenza-induced pathogenicity (Camp and Jonsson, 2017; George et al., 2021), as well as innate immune cells other than macrophage in the H7N9/H9N2-infected lungs and BAL fluids. Our analysis of lung transcriptomics did suggest a potential early lung enrichment of neutrophiles in response to H9N2 infection, evidenced by CXCR2, the main receptor mediating the lung migration of neutrophils during influenza infection, and three major neutrophil-attracting chemokines, namely CCL3, CXCL-1, and CXCL2, which all exhibited a pattern consistent with an early induction by H9N2 infection compared to H7N9 infection (data not shown). The illustration of the complete landscape of changes in innate immune response to H9N2 versus H9N2, as well as the dissection of the functional contributions of individual immune cell types to such response, clearly warrant future investigation.

We also probed the dynamics of the T cell immune response reacting to H9N2 and H7N9 infections. The results showed a more robust CD8+ T cell response in the lung induced by the H7N9 infection than H9N2 infection. Importantly, for the H7N9 infection, such T cell response showed a pattern of gradual increase with a peak attained on day 2 post infection. Our previous studies of hospitalized H7N9 patients revealed the virus resolution and survival depends on a diversity of response mechanisms, wherein an early prominent H7N9-specific CD8(+) T-cell responses is translated into a shorter discharge time and increased survival. This rapid CD8(+) T cell response is potentially derived from cross-reactive memory T cells deposited by prior influenza exposure (Wang et al., 2015). Utilizing a sequential infection mouse model, we have recently demonstrated that lung-resident influenza-specific CD8+ T cells are promptly activated and secrete IFN-γ in response to influenza re-exposure, thereby establishing a tissue-wide antiviral state for effective viral inhibition (Jiang et al., 2022). In the current study, the mice were naïve to influenza and thus the increased number of CD8+ T cells in the H7N9-infected lung must be due to recruitment of circulating CD8+ T cells. Whether such strong T cell response, which occurred at relatively late time points after infection, functions in viral clearance, contributes to the lung histopathology, or both remains an open question and we speculate the dual effects would be more likely. In this regard, the measurement of the functionality of CD8+ T cells, as well as the assessment of their possible redistribution among different tissue compartments, will be informative to further define the role of a primary T cell response in the anti-H7N9 host immunity. They are definitely worthy of future investigation.

Finally, we performed a co-infection experiment to examine our hypothesis that the elicitation of an early innate immunity is critical for protection against influenza virus infection. We could show that co-inoculation of H9N2 did not enhance the lethality and pathogenicity of the host, but rather reduced the pathogenicity of H7N9 infection. An even better protective effect was observed with the PR8 virus. The lesser efficacy of H9N2-mediated protection against H7N9 versus PR8 might be due to a relatively higher replicative capacity of the H7N9 virus. Given that H7N9 and PR8 viruses express very different HA and NA surface proteins, the ability of H9N2 co-infection to protect mice against the challenge of a heterologous influenza virus is consistent with a mechanism primarily mediated by innate immunity, as we proposed. Taken together, we think that the lesser pathogenicity of H9N2 in the mouse model has dual mechanism: first, it is intrinsically attenuated when infecting mammalian cells and our previous study has pinpointed such defect to the lack of mammalian adaptation of its internal genes, most likely its polymerases genes (Zhang et al., 2020); secondly, it triggers an earlier innate immune response in infected lung as compared to H7N9, resulting in more effective viral inhibition.

In summary, we presented here a comparative analysis of immune responses to the H7N9 virus versus the H9N2 virus. This analysis, together with a following demonstration of the protection against H7N9 and PR8 challenge by H9N2 co-infection, brings a new perspective to the interaction between host immunity and influenza evasion. That is, the induction of early innate immune response, as seen with in H9N2 infection but absent in H7N9 infection, is the first but also potentially a primary step toward an effective viral inhibition. Without this step, adaptive immunity could be responsible for viral clearance; however, a sustained CD8+ T cell response may leave the host at higher risk of lung immunopathology. There are some important questions warranting future investigation. For example, what is the mechanism mediating H7N9’s escape from trigging an early innate immune response? Is there any specific viral factor involved? An equally important question is to identify the pathogen-associated molecular pattern (PAMP) pathway(s) responsible for the detection of H9N2 infection. It should be noted that the importance of such a pathway would not be unraveled in the case of H7N9, where a viral evasion mechanism might evolve. Furthermore, is avoiding early innate immunity a general mechanism utilized by other influenza viruses that cause severe diseases? Conversely, a comparative measurement of pulmonary specific compliance and the composition of bronchoalveolar fluid is a worthy effort that has the potential to provide new clues into the H7N9-caused lung pathology and the underlying causes. These future investigations would certainly help us further understand the complex host-influenza interactions, thereby providing a new theoretical basis for development of new therapeutics for treatment of influenza-related human diseases.

## Data availability statement

The original contributions presented in the study are included in the article/supplementary material. Further inquiries can be directed to the corresponding author/s.

## Ethics statement

The animal study was reviewed and approved by Shanghai Public Health Center Local Ethical Committee.

## Author contributions

XZ and JX conceived and designed this project and supervised the experiments. CZ and YY performed the IHC and IF experiments. WF performed extraction of total RNA and microarray analysis. MZ performed virus titer determination. YH, LZ, SY and LJ performed the *in vivo* experiments. All authors contributed to the article and approved the submitted version.

## Funding

This work was supported by the National Natural Science Foundation of China (81672018,82071788, 82101846) , the Intramural Funding from Shanghai Public Health Clinical Center (KY-GW-2019-16, KY-GW-2021-03), Shanghai Science and Technology Program (20Y11900500), and Sichuan Science and Technology Program (2021YFS0404 and 2021ZYD0082).

## Acknowledgments

We thank Professor Zejun Li of Shanghai Veterinary Research Institute, Chinese Academic of Agricultural Sciences for providing virus.

## Conflict of interest

The authors declare that the research was conducted in the absence of any commercial or financial relationships that could be construed as a potential conflict of interest.

## Publisher’s note

All claims expressed in this article are solely those of the authors and do not necessarily represent those of their affiliated organizations, or those of the publisher, the editors and the reviewers. Any product that may be evaluated in this article, or claim that may be made by its manufacturer, is not guaranteed or endorsed by the publisher.
